# Twist Angle Tuning of Moiré Exciton Polaritons in van der Waals Heterostructures

**DOI:** 10.1021/acs.nanolett.2c01175

**Published:** 2022-05-20

**Authors:** Jamie M. Fitzgerald, Joshua J. P. Thompson, Ermin Malic

**Affiliations:** † Department of Physics, Chalmers University of Technology, SE-412 96 Gothenburg, Sweden; ‡ Department of Physics, Philipps University, 35037 Marburg, Germany

**Keywords:** transition metal dichalcogenides, moiré excitons, polaritons, van der Waals hetero-bilayers

## Abstract

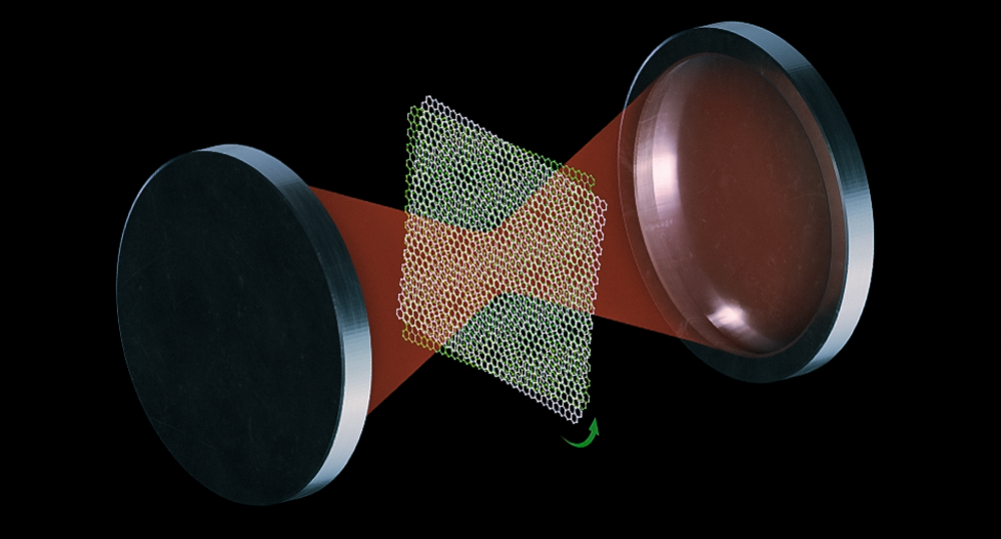

Twisted atomically thin semiconductors are characterized by moiré excitons. Their optical signatures and selection rules are well understood. However, their hybridization with photons in the strong coupling regime for heterostructures integrated in an optical cavity has not been the focus of research yet. Here, we combine an excitonic density matrix formalism with a Hopfield approach to provide microscopic insights into moiré exciton polaritons. In particular, we show that exciton-light coupling, polariton energy, and even the number of polariton branches can be controlled via the twist angle. We find that these new hybrid light-exciton states become delocalized relative to the constituent excitons due to the mixing with light and higher-energy excitons. The system can be interpreted as a natural quantum metamaterial with a periodicity that can be engineered via the twist angle. Our study presents a significant advance in microscopic understanding and control of moiré exciton polaritons in twisted atomically thin semiconductors.

Two monolayers of transition metal dichalcogenides (TMDs) can be vertically stacked to form a type-II heterostructure.^1−4^ MoSe_2_/WSe_2_ is a typical example, which exhibits a large band offset such that electronic hybridization at the K point is negligibly small,^5^ leading to well-defined intralayer and interlayer exciton states.^6^ The former have a large oscillator strength and manifest as a visible signal in absorption spectra. It has also been demonstrated that artificial moiré superlattices can be formed by engineering a finite twist angle between TMD layers.^7−9^ One consequence is the emergence of multiple flat exciton minibands, significantly modifying the optical emission^10,11^ and absorption spectra^8,12−14^ of TMD hetero-bilayers. In a twisted geometry, the electronic band energies at the K point vary periodically in space reflecting the local atomic registry of neighboring layers. If the resulting moiré potential is deep enough, low-energy excitons can be trapped, and hopping between adjacent supercells is strongly suppressed.^13,15−18^ Excitons in monolayer TMDs have a large oscillator strength and binding energy, making them suitable for integration into optical microcavities for exploration of the strong coupling regime.^19^ Here, the light-matter coupling strength exceeds dissipation in the material and radiative decay from the cavity.^20^ In this regime, the role of exciton polaritons has been explored theoretically^21−23^ and observed experimentally for TMDs placed in conventional dielectric Fabry–Perot cavities,^24,25^ as well as using Tamm-plasmon photonic microstructures,^26^ subwavelength-thick photonic crystals,^27^ plasmonic lattices,^28^ and nanodisks.^29^ Unique properties of TMDs have been exploited to demonstrate valley polarized polaritons,^30,31^ trion polaritons,^32^ and Bose–Einstein condensation.^33^

Little is known about the strong coupling physics of twisted heterostructures and the impact of moiré superlattices. Recently, there was a first experimental demonstration of polaritons in a twisted WS_2_/MoSe_2_ hetero-bilayer,^34^ where the density dependence of the localized moiré polaritons revealed a strong nonlinearity due to the exciton blockade. For MoSe_2_/WSe_2_ hetero-bilayers specifically, only the weak-coupling regime has been explored so far.^35,36^ In this work, we develop a microscopic model of moiré exciton polaritons, focusing on the twist angle as a new knob to control their optical response. We study the strong coupling between *intralayer* moiré excitons and cavity photons in a twisted AA-stacked MoSe_2_/WSe_2_ hetero-bilayer placed in the center of a Fabry–Perot cavity, cf. Figure 1a. This exemplary heterostructure has been the focus of recent studies.^10,13,37^ A material-realistic combined Wannier–Hopfield model reveals multiple distinct branches of moiré exciton polaritons and enables the first microscopic insights into the dispersion, absorption, hybridization, and localization of moiré polaritons over a wide range of twist angles.

**Figure 1:**
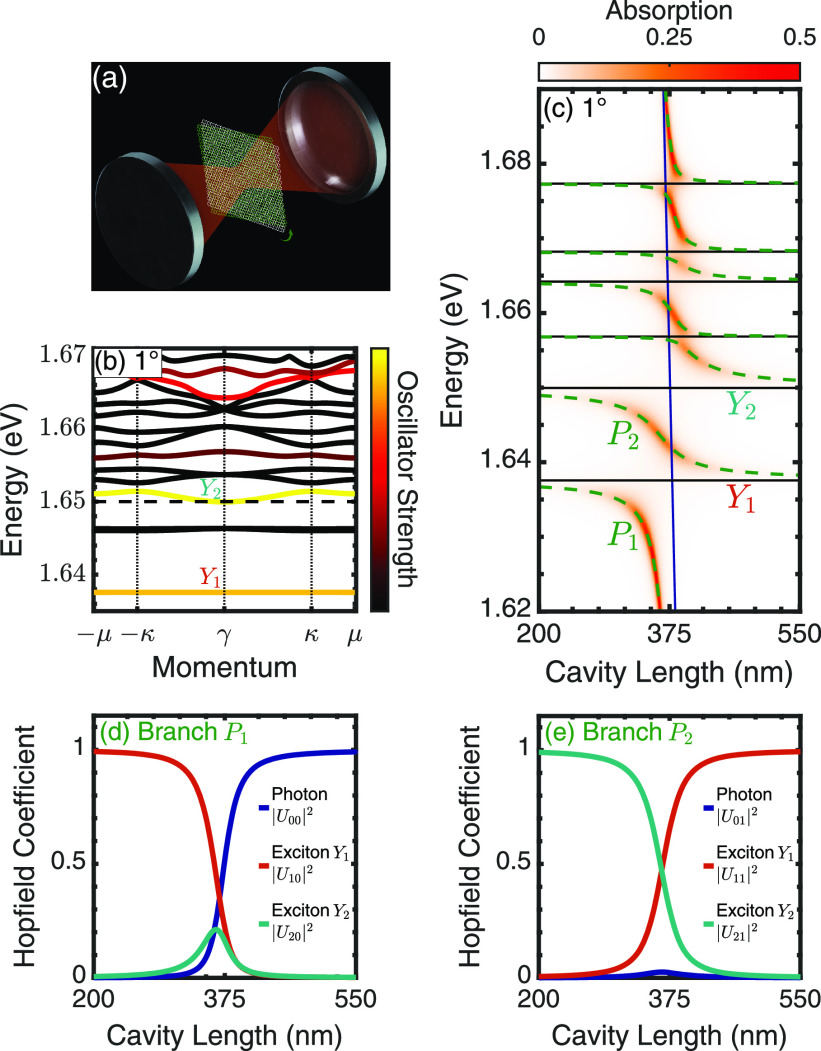
(a) Illustration of a hetero-bilayer placed in the center of a Fabry–Perot cavity. (b) Moiré exciton minibands for the intralayer exciton at a 1° twist angle. Color coding is proportional to the oscillator strength. The black dashed line indicates the MoSe_2_-based exciton energy in the absence of moiré effects. (c) Cavity length dependence of moiré exciton polariton bands (dashed green lines) overlaid on the absorption for a 1° twist angle. Bare exciton (cavity) energies are indicated by the black (blue) lines. Absolute square of the Hopfield coefficients, showing the photonic (*U*_0*n*_) and excitonic contributions (*U*_1*n*_ and *U*_2*n*_) to the polariton branch (d)  and (e)

The moiré exciton Hamiltonian is modeled within the tight-binding approximation by assuming that the moiré potential stems from the interaction of the neighboring layer’s d-orbitals, which are the major orbital contribution at the K points.^13,38^ The model is valid in the continuum limit of small angles.^15,16,39^ The band structure of the MoSe_2_-based intralayer exciton at a twist angle of 1° is shown in Figure 1b. Below the unperturbed 1s intralayer exciton energy (i.e., neglecting moiré effects and denoted by the black dashed line), three flat bands are found, indicating that these excitons are localized within the moiré potential. Excitons at the γ point (center of the mini Brillouin zone (mBZ)) are located within the light cone; however, because of symmetry reasons some of these branches interact very weakly with light, behaving as dark excitons. This is dictated by the value of the moiré exciton wave function at the γ point, . The radiative coupling of the νth exciton is given by , where *ℏ*γ^(*X*)^ is the coupling in the absence of moiré effects. The radiative coupling is shared between moiré excitons at the γ point, with the total oscillator strength conserved, and exhibits a twist-angle dependence via the moiré wave functions. The different exciton sub-bands in Figure 1b are color coded by the magnitude of the oscillator strength at the mBZ center. The bands labeled  and  correspond to the two energetically lowest bright excitons. As we are interested in absorption spectra, dark excitons are not further considered.

Placing the hetero-bilayer in a cavity leads to an enhanced resonant coupling between the excitons and cavity photons. The corresponding Hamiltonian in a rotating-wave approximation reads
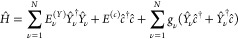
1where  are the moiré exciton field operators (energies) and  are the cavity-photon field operators. It can be converted to the polariton basis via a Hopfield diagonalization,^40^ where the *n*th-band polariton field operator is expressed as . The expansion coefficients *U*_*mn*_ are known as Hopfield coefficients and measure the contributions of the *m*th constituent photon/exciton to the *n*th polariton state. In particular, *U*_0*n*_ quantifies the photonic nature of the *n*th polariton. The coupling can be determined analytically for the specific case of a thin excitonic material placed in the center of a high-quality symmetric Fabry–Perot cavity at frequencies close to the cavity resonance^20^
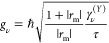
2where *r*_m_ is the end-mirror reflectivity, τ = *L*/*c* the one-way photon travel time in the cavity, and *L* is the cavity length. Note that the coupling will change with the twist angle via the dependence on the radiative coupling. Further details on all the theoretical methods used in this work are provided in the Supporting Information.

One strategy to explore the strong coupling regime is to detune photon and exciton energies by changing the cavity length.^25^ We consider only the lowest-energy cavity mode with a vanishing in-plane momentum. Figure 1c shows the polariton energy as a function of cavity length for a fixed twist angle of 1°. Because of the large coupling of intralayer excitons to light, it is necessary to include both intralayer excitons located in the MoSe_2_ (1.65 eV in absence of moiré effects) and WSe_2_ layer (1.75 eV). There are also two interlayer configurations, in particular, a low-energy interlayer exciton^6,13^ at 1.35 eV, but their small oscillator strength makes them challenging to utilize for strong coupling physics. For simplicity, we will concentrate our analysis on the MoSe_2_-based intralayer excitons. The energy of the polariton branches is indicated by green-dashed lines in Figure 1c and is overlaid on a color map of the polariton absorption. A strong coupling between the cavity photon (blue line) and the six bright moiré excitons (flat black lines) is indicated by an avoided crossing of the resulting polariton dispersion near the points of intersection.

The small energy separation between moiré exciton bands (∼10 meV at 1°) is on the same order of magnitude as the coupling strength between the individual moiré excitons and the cavity photon. This leads to a set of seven polariton branches including five middle branches of a mostly excitonic nature and an upper and lower polariton branch with a strongly cavity length-dependent hybrid light-exciton nature. The lower polariton branch, , can be readily understood: for small cavity lengths (*L* < λ/2, where λ is the cavity-photon wavelength corresponding to the  exciton energy), the cavity is off-resonant, and the polariton branch follows the bare energy of the  exciton. There is an avoided crossing near the intersection point around 378 nm due to the hybridization between the exciton and the photon. At longer cavity lengths, the polariton becomes increasingly light-like.

This inferred behavior is confirmed by studying the Hopfield coefficients, cf. Figure 1d, where the contribution of the photon, and the  and  excitons is shown. They reveal that  also has a significant contribution from  close to the avoided crossing. This photon-mediated exciton hybridization occurs for polaritonic systems with multiple energetically closely spaced excitons (relative to the coupling strength).^41^ Interestingly, the cavity can strongly mix excitons without the resulting polariton possessing a strong photonic component, as nicely illustrated by the  polariton in Figure 1e. Around the avoided crossing, the polariton is an almost equal combination of the  and  exciton, with only a small photonic contribution.

It is instructive to explore the polariton absorption, which unambiguously demonstrates strong coupling via the Rabi splitting.^42^ It can be readily calculated using the T-matrix method;^20^ however, it is fruitful to combine the Hopfield approach with the input-output formalism,^43^ which works well in the limit of high-Q cavities (i.e., |*r*_m_| ≈ 1). Using this approach, we quantize separately the internal cavity mode and the external radiation fields, which are weakly coupled via the finite transmission of the end mirrors. Then, we convert to a polariton basis and find the Heisenberg–Langevin equations, using the Markov approximation for the mirror coupling. These are are then complemented with the input–output relations, which relate incoming and outgoing fields at each port.^43^ Further, we include a phenomenological model of loss by coupling each moiré exciton to its own phonon bath. This leads to a consistent microscopic description of the radiative decay rate, material loss, and the coupling of the polaritons to external fields. In the macroscopic limit, this approach is equivalent to the coupled-mode theory that is commonly used in photonics.^44^ In the limit of a small scattering loss, Γ, relative to energy spacing between excitons, we obtain an Elliot-like expression
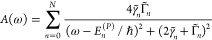
3with an effective radiative coupling  and scattering loss  for the *n*th polariton. The polaritonic Elliot formula is centered on the polariton energy  with a width determined by the sum of the effective nonradiative and radiative couplings. The latter plays the same role as the radiative coupling in the usual excitonic Elliot formula^45^ and describes how polaritons couple to the external ports. Intuitively, it is equal to the bare-cavity decay rate, κ = *cT*_*m*_/4*L*, scaled by the Hopfield coefficient describing the photonic contribution of the *n*th polariton, |*U*_0*n*_|^2^. The cavity properties enter directly through both the length, *L*, and the transmission of the end mirrors, *T*_*m*_. The latter results from the fact that light may only couple to outside ports through the end mirrors. Similarly, the effective scattering loss is dependent on the total excitonic contribution to the polariton via the factor . This reflects that all nonradiative decay channels are included via excitons in our model. Equation 3 reveals that the absorption is dictated by a compromise between the excitonic and photonic contribution. In analogy to a bare excitonic system, the maximum possible absorption is 0.5 due to the mirror symmetry of the system^46^ and occurs when total photonic decay (in this case through both ports) is equal to exciton scattering loss, i.e., . The value of |*U*_0*n*_|^2^ at which this polaritonic critical-coupling condition is met is dictated by the balance between the exciton scattering rate and the cavity decay rate. This demonstrates the utility of the polaritonic Elliot formula for designing cavities that efficiently couple energy into polaritons and providing microscopic intuition for experimental observables.

Taking the  polariton branch as an example, for small *L* the cavity will not allow light in, and no absorption can take place. This is captured by a vanishing *U*_0*n*_ and hence , in eq 3. For large *L*, *U*_0*n*_ limits toward 1, and the contribution of the exciton scattering loss vanishes . Here, we recover the absorption of a bare cavity (which is zero in our model). For intermediate cavity lengths, close to the avoided crossings, we observe the largest absorption (cf. Figure 1c). For the parameters used in this work (Γ = 1 meV, |*r*_m_| = 0.99), peak absorption of 0.5 is found at |*U*_0*n*_|^2^ = 0.16, which corresponds to a cavity length of 364 nm (see Supplementary Figure S1). In contrast, polariton  is weakly photonic (small |*U*_0*n*_|^2^; see Figure 1e) even near the avoided crossing region and hence has a lower absorption compared to the  branch.

A unique characteristic of low-energy moiré excitons is localization within the moiré potential for small twist angles.^13,15,16^ In contrast, the cavity photon is completely delocalized in the transverse plane, and the polariton is expected to at least partially inherit this property. One means of inferring the degree of localization is via the group velocity,^47^, and the effective mass , where **k**_∥_ is the polariton in-plane momentum. For near-flat bands, i.e., the localized  exciton in Figure 1b, the group velocity vanishes over an extended region of the mBZ, and the exciton branch is characterized by a large effective mass.

To this end, we perform a study for TM-polarized oblique cavity photons with a finite in-plane momentum, concentrating on the  branch for different detuning of the cavity photon and exciton energy at **k**_∥_ = 0, cf. Figure 2. The large effective mass of the moiré exciton, *m*^(*Y*)^ = 77*m*_0_, relative to the effective photon mass, *m*^(*c*)^ = *E*^(*c*)^(**k**_∥_ = 0)/*c*^2^ = 3 × 10^–6^*m*_0_, means that the exciton branch is essentially flat (red dashed line in Figure 2) relative to the cavity dispersion . By detuning the cavity photon and exciton energy via the cavity length, the mixing of the  exciton with the cavity photon and higher-energy excitons can be modified. This can be understood using the Hopfield approach (see Supporting Information) yielding for the inverse effective mass of the polariton
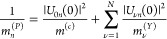
4

**Figure 2:**
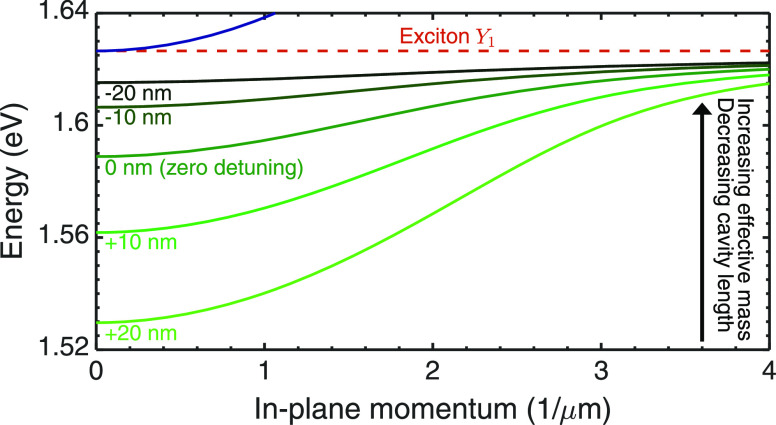
Detuning study of the lowest-energy TM-polarized moiré exciton polariton dispersion for a 1° twisted hetero-bilayer. Exciton (cavity photon at zero detuning) energy is shown by the dashed red (solid blue) line for comparison. The curvature and hence the group velocity and the effective mass of the polariton are drastically altered by tuning the cavity resonance relative to the  exciton energy. Very similar results are found for TE polarization around zero in-plane momentum.

The increasing photonic character of  for larger cavities, revealed by the Hopfield coefficients in Figure 1d, manifests as an increased curvature in Figure 2, and hence a smaller effective mass. Consequently, the effective mass can be tuned over a staggering seven orders of magnitude, from *m*^(*Y*)^ → *m*^(*c*)^. Equation 4 reveals that one needs to detune the cavity resonance very far from the exciton energy to achieve a polariton mass comparable to the exciton mass since for comparable Hopfield coefficients the term ∝ 1/*m*^(*c*)^ dominates. As a consequence, when the polariton exhibits even a small photonic character, it tends to be delocalized over many moiré unit cells.

The lattice mismatch in MoSe_2_/WSe_2_ hetero-bilayers is very small, and thus the moiré potential is strongly dependent on the twist angle. The twist-angle dependence of moiré polaritons follows from (i) the energy detuning between the cavity photon and each moiré exciton changes with the twist angle, and (ii) the oscillator strength accumulates with increasing twist angle into the lowest-energy exciton at the expense of all the others, cf. Figure 3a for the coupling strength, *g*_ν_, of the three lowest exciton branches. As the twist-angle increases, *g*_1_ limits to the untwisted value, *g*^(*X*)^ = 42.6 meV.

**Figure 3:**
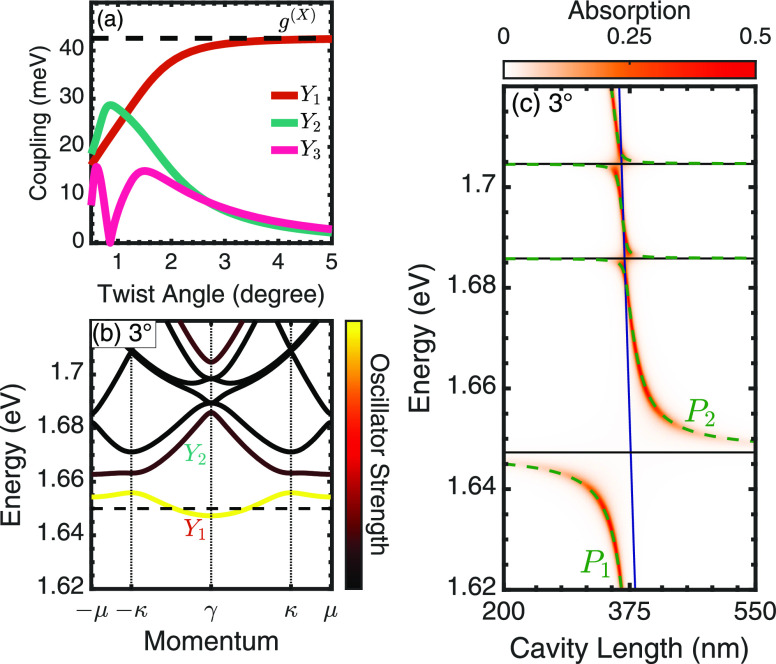
(a) Coupling strength of the three lowest-energy moiré excitons as a function of the twist angle. The black dashed line shows the radiative coupling of the intralayer exciton in the absence of moiré effects. (b) Moiré exciton minibands for the intralayer exciton at a 3° twist angle. Color coding is proportional to the oscillator strength. (c) Cavity length dependence of the moiré polariton bands (dashed green line) overlaid on the absorption for a twist angle of 3°.

To illustrate the twist-angle dependence, we repeat the cavity length sweep for an angle of 3°. At this larger angle, there is significant hopping of excitons between moiré supercells, which is reflected by shallow, near-parabolic energy dispersion of the  exciton, cf. Figure 3b. The accumulation of oscillator strength in  leads to a larger Rabi splitting when compared to the 1° case, cf. Figure 3c. The coupling strength for  is nearly doubled from 24 meV at 1° to 41 meV at 3°. The presence of higher-energy moiré excitons is visible in the much weaker avoided crossings close to 1.68 and 1.70 eV.

Now, we investigate the polariton dispersion and absorption for a range of twist angles between 0.5° and 4.5°. The cavity energy is twist-angle independent (flat blue line) and tuned to the untwisted exciton energy of 1.65 eV (*L* = λ/2 = 374 nm). In contrast, the moiré exciton energies have a clear twist-angle dependence, cf. Figure 4a. In particular, the  exciton shifts by 14 meV as the twist angle is tuned from 0.5° to 3°. We find an abundance of bright excitons, which decrease in number for an increasing twist angle.^13^ At larger angles, the only notable absorption arises from the lowest  branch due to the accumulation of oscillator strength.

**Figure 4:**
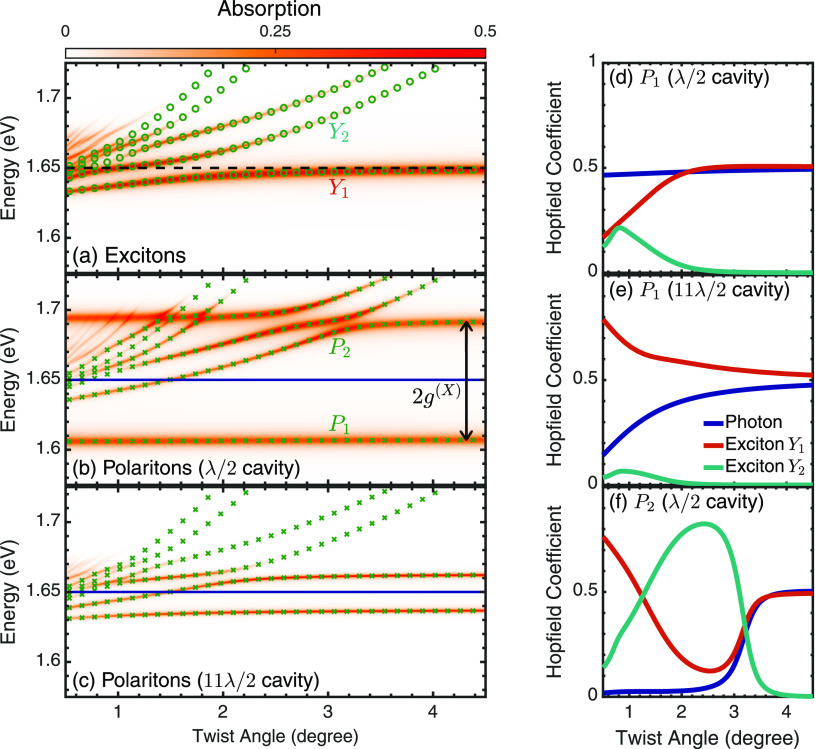
(a) Twist-angle dependence and absorption of the intralayer moiré excitons in the absence of a cavity. The exciton energy is shown by the green circles. Twist-angle dependence and absorption of moiré polaritons (green crosses) in a (b) λ/2 = 374 nm and (c) 11λ/2 = 4120 nm cavity. The cavity mode energy is shown by the flat blue line and is equal to the 1s intralayer exciton energy in the absence of moiré effects (black dashed line in (a)). Absolute square of the Hopfield coefficients for the  polariton branch in a (d) λ/2 and (e) 11λ/2 cavity, and (f) the  polariton branch in a λ/2 cavity.

Figure 4b shows the polariton dispersion and absorption for the hetero-bilayer placed within a cavity. The strong coupling leads to the almost completely twist-angle independent  polariton branch that is red-shifted by *g*^(*X*)^ relative to the cavity mode energy. The polariton energy shifts less than 1 meV over the angle range of 0.5° → 3°. At larger twist angles (≳ 3°), this is expected due to the oscillator strength accumulating into  (Figure 3a), which has a near twist-angle independent energy at these angles (Figure 4a). This manifests as a twist-angle independent  and  in the strong coupling regime, and the splitting between the two limits to the untwisted value of 2*g*^(*X*)^ = 85 meV (indicated by the black arrow). The behavior of the polariton  at small angles is puzzling, as the analysis of the Hopfield coefficients (Figure 4d) shows that it has a sizable contribution from  and , which both have a strong twist-angle dependence for small angles. The photonic contribution, on the other hand, is a nearly constant 0.5 over all twist angles, cf. Figure 4d. This leads to the almost twist-angle independent absorption apparent in Figure 4b, cf. eq 3.

To elucidate further, if the coupling strength is decreased by extending the cavity length (see eq 2), then the  polariton starts to recover a similar twist-angle dependence as the  exciton, cf. Figure 4c for a *L* = 11λ/2 = 4120 nm cavity (energy shift of 5 meV over the range 0.5° → 3°). The polariton twist-angle dependence can be understood as a compromise between (i) the exciton-cavity detuning, i.e., blue-shift of  and hence  with the twist angle (Figure 4a), and (ii) coupling strength, i.e., larger splitting due to accumulating oscillator strength with increasing twist angle (Figure 3a), which leads to a red-shift of  due to the increased Rabi splitting. For the larger coupling, the photon can effectively couple to the  exciton at all twist angles and thus possesses a large photonic component of nearly 50% for all angles (Figure 4d). In contrast, the weaker coupling for the 11λ/2 cavity means that at small angles,  has a larger excitonic component and consequently inherits a proportion of the twist-angle dependence of , cf. Figure 4e.

The  polariton shows a stronger twist-angle dependence than  for both cavity lengths at small angles, shifting about 47 meV from 0.5° to 3° for the λ/2 cavity, before plateauing like . Inspection of the Hopfield coefficients in Figure 4f reveals that this arises from the large contribution of  at intermediate angles. As the twist angle increases above 3°, the photonic and  contributions grow to 0.5 each, explaining the growing angle independence. It is interesting to observe a weaker twist-angle dependence of  for the 11λ/2-cavity, cf. Figure 4c. There is now a energy shift of 23 meV over the range 0.5° → 3°, illustrating that modifying the cavity length can reduce or increase the twist-angle dependence of different polariton branches. At large twist angles, all other higher-energy polariton branches tend to a linear dependence, following the behavior of bare excitons, and exhibit a negligible absorption due to the vanishing oscillator strength and large detuning.

This work sheds light on the optical response of intralayer moiré exciton polaritons in twisted van der Waals hetero-bilayers integrated within a Fabry–Perot cavity. Specifically, we have shown that the small spacing between moiré excitons and the large coupling with light inherent to TMDs lead to a distinct set of polariton branches, which can consist of multiple excitons due to photon-induced hybridization. Exploiting the Hopfield approach allows us to characterize the energy, absorption, and localization of these unique polaritons. The rich twist-angle and cavity dependence succinctly exemplifies their dual light--matter character. Progress in stacking technologies with controllable small-angle increments^48^ and tunable cavity length^25^ could allow for experimental investigation of the predicted intriguing properties of moiré polaritons. Our theory also lays the foundation for future work; for instance, the phenomenological inclusion of loss within the polaritonic Elliot formula could be greatly improved upon by explicitly considering polariton scattering with phonons.^49,50^

## Supporting Information Available

Details of the excitonic density matrix formalism and small-angle effective continuum model, as well as parameters used for calculations. Comment on photon-induced interlayer hybridization. Details on the alternative transfer matrix method for modeling exciton polaritons. Detailed derivation of the polaritonic Elliot formula (eq 3) using the Heisenberg–Langevin equations. Discussion of the polaritonic critical-coupling condition. Derivation of the polariton effective mass for an N-exciton system (eq 4) (PDF)
