# Supplementary material for: Twist Angle Tuning of Moiré Exciton Polaritons in van der Waals Heterostructures
Source: Nano Lett. 2022 May 20;22(11):4468–74. doi: 10.1021/acs.nanolett.2c01175 (PMC9185750; doi:10.1021/acs.nanolett.2c01175)
Supplement: Supplementary file 1 — nl2c01175_si_001.pdf [file nl2c01175_si_001.pdf]

Supplementary Information
Twist Angle Tuning of Moiré Exciton Polaritons in van der Waals Heterostructures

Jamie M. Fitzgerald,[*,†] Joshua J. P. Thompson,[‡] and Ermin Malic[†,‡]

†*Department of Physics, Chalmers University of Technology, SE-412 96 Gothenburg, Sweden*

‡*Department of Physics, Philipps University, 35037 Marburg, Germany*

E-mail: jamief@chalmers.se

S.1 Theoretical Methods

S.1.1 Modelling Moiré excitons

To model the excitonic behaviour of a TMD heterobilayer, we first solve the bilayer Wannier equation for a given layer configuration of the constituent electron and hole, taking into account the screening effect of the immediate dielectric environment.[1] This allows for a transformation into an effective single-particle Hamiltonian using an exciton basis, $\hat{H} = \sum_{\mathbf{Q}} E^{(X)}_{\mathbf{Q}} \hat{X}^{\dagger}_{\mathbf{Q}} \hat{X}_{\mathbf{Q}} + \sum_{\mathbf{Q},\mathbf{q}} \mathcal{M}_{\mathbf{q}} \hat{X}^{\dagger}_{\mathbf{Q}+\mathbf{q}} \hat{X}_{\mathbf{Q}}$, where $\hat{X}_{\mathbf{Q}}$ is the exciton field operator at the center-of-mass momentum $\mathbf{Q}$, and $\mathcal{M}_{\mathbf{q}}$ the matrix dictating the mixing of excitons by the moiré potential (see subsection S.1.2). Next, a zone-folding approach is applied, where $\mathbf{Q}$ is restricted to the mini Brillouin zone (mBZ). A projection to a moiré exciton basis gives,[2] $\hat{H} = \sum_{\nu,\mathbf{Q}} E^{(Y)}_{\nu}(\mathbf{Q}) \hat{Y}^{\dagger}_{\nu,\mathbf{Q}} \hat{Y}_{\nu,\mathbf{Q}}$, with $\mathbf{Q} \in$ mBZ and $\hat{Y}_{\nu,\mathbf{Q}} = \sum_{i,j} \mathcal{C}_{\nu,i,j}(\mathbf{Q}) \hat{X}_{\mathbf{Q}+i\mathbf{g}_0+j\mathbf{g}_1}$, where the vectors $\mathbf{g}_0$ and $\mathbf{g}_1$ span the mBZ. The moiré exciton wavefunction and energy are given

by $\mathcal{C}_{\nu,i,j}(\mathbf{Q})$ and $E_\nu^{(Y)}(\mathbf{Q})$, respectively. We take a rigid-lattice approximation and do not consider atomic reconstruction, which has been shown to be important for $MoSe_2/WSe_2$ heterostructures at twist angles smaller than about 1°.[3] While this significantly changes the shape of the potential well seen by localised excitons, we believe that the qualitative features of the exciton polariton physics described here remain the same. It is also important to note that while our model assumes perfect crystal lattices, moiré excitons have been demonstrated in imperfect real-life heterostructures.[4] Defects offer another interesting route to localized excitons.[5,6] Moiré excitons can be distinguished from defect-localized excitations by their twist-angle dependence and the g-factors in presence of magnetic fields.[4] We expect the main results of our study, in terms of the predicted twist-angle dependence of moiré exciton polaritons, to be valid even in imperfect samples.

S.1.2 Moiré potential

Here we briefly recap the theory developed in Ref. 2 which we utilise in this work. The moiré potential describes the twist-angle dependent electrostatic potential felt by charge carriers in one layer induced by neighbouring atoms in the other layer. Using the monolayer eigenstates to expand the moiré contribution to the bilayer Hamiltonian gives $\hat{H}_\mathrm{M} = \sum_{l,\lambda,\mathbf{kk}'} \langle l\lambda\mathbf{k}|\hat{V}_{\tilde{l}}|l\lambda\mathbf{k}'\rangle\, \hat{a}^\dagger_{l\lambda\mathbf{k}}\hat{a}_{l\lambda\mathbf{k}'}$, where, $l = \{0,1\}$ is the layer index, $\tilde{l} = 1-l$, V_l is the effective electrostatic potential created by layer l, and $\lambda = \{\mathrm{v},\mathrm{c}\}$ is the band index. The Bloch wavefunction for the lth layer is written within a tight-binding approach using an envelope-function approximation around the K-points. The resulting matrix element, $V_{l,\lambda}(\mathbf{q}) = \langle l\lambda\mathbf{k}+\mathbf{q}|\hat{V}_{1-l}|l\lambda\mathbf{k}\rangle$, is then expressed within a two-site approximation, split into a sum of atomic contributions, and finally a first-shell approximation is taken[7]

$$V_{l,\lambda}(\mathbf{q}) = \mathcal{V}_{l,\lambda}\sum_{n=0}^{2} \exp\left[iC_3^n\left(\mathbf{G}_l^0 + \mathbf{G}_{\tilde{l}}^0\right)\cdot\mathbf{d}/2\right]\delta_{\mathbf{q},\mathbf{g}_n}, \tag{S.1}$$

where $\mathbf{d}$ is the lateral displacement between the two TMD layers. The vectors $g_n = C_3^n \left(\mathbf{G}_{\tilde{l}}^0 - \mathbf{G}_l^0 \right)$ span the mBZ reciprocal to the periodic moiré pattern formed from the twisted lattices. The first-shell approximation involves restricting the contribution of reciprocal lattice vectors to $C_3^n \mathbf{G}^0$ $(n = 0, 1, 2)$. This approximation preserves the C_3 symmetry of the moiré potential, and is the same form as phenomenological formulas used in previous works.[8–10] The potential $\mathcal{V}_{l,\lambda}$ quantifies the strength of the potential and is obtained as material-specific external input parameter from first-principles calculations, see Ref. 2.

After transforming to the exciton basis, the moiré matrix element can be written as[2,11]

$$\mathcal{M}_{ll',\mathbf{q}} = V_{l,c}(\mathbf{q})\mathcal{F}_{ll'}(\beta_{ll'}\mathbf{q}) - V_{l',v}(\mathbf{q})\mathcal{F}_{ll'}^*(\alpha_{ll'}\mathbf{q}), \quad \text{(S.2)}$$

which describes the asymmetry between how the moiré potentials affects the constituent electron and hole of the exciton for a given layer configuration. Furthermore, it represents the mixing of excitons with momenta that differ by a reciprocal superlattice vector, $\mathbf{g}_n$, as enforced by the delta function in equation S.1. The factors $\alpha_{ll'}(\beta_{ll'}) = m_{c,l}(m_{v,l'})/(m_{c,l}+m_{v,l'})$ describe the impact of the electron and hole masses, and $\mathcal{F}_{ll'}(\mathbf{q}) = \sum_{\mathbf{k}} \Psi_{ll'}^*(\mathbf{k})\Psi_{ll'}(\mathbf{k}+\mathbf{q})$ is the excitonic form factor, which itself depends on the excitons wavefunctions returned by solving the Bilayer Wannier equation.

S.1.3 Bilayer Wannier equation

Solving the bilayer Wannier equation gives access to the 1s exciton wavefunction $\Psi_{ll'}(\mathbf{r})$, and its excitonic binding energy for a given layer configuration. The screened Coulomb potential is modelled as a generalised Keldysh potential for two aligned and anisotropic slabs.[1] The heterostructure is hBN encapsulated ($\epsilon_{\text{sub}} = 4.5$), and the dielectric constants used for the TMD layers are taken from Ref. 12. The spectral position of the 1s exciton, $E^{(X)}(0)$, is fixed to 1.65 eV (1.75 eV) for the $MoSe_2$(WSe_2)-based intralayer exciton, which have been extracted from photoluminescence measurements.[13,14] The dielectric constant of the media

surrounding the TMD heterostructure will impact the screened Coulomb interaction and hence alter the exciton energy and coupling strength to light. Therefore, the choice of encapsulating material can be used an additional means to tune TMD exciton polaritons.[15]

S.1.4 Exciton-light coupling

The radiative coupling of a bilayer exciton in an untwisted and lattice-matched heterostructure, i.e. with no moiré effects, has the form[16]

$$\gamma^{(X)} = \frac{e^2 \left|\Psi(\mathbf{r}=0)\right|^2 \left|\mathbf{p}_{cv} \cdot \mathbf{e}_\sigma\right|^2}{2m_e^2 \epsilon_0 n_{bg} c E^{(X)}(0)}, \tag{S.3}$$

where n_{bg} is the background cavity reflectivity, and $\mathbf{e}_\sigma$ is a polarization vector of the light. For intralayer configurations, the optical matrix element for transitions near the K point reduces to the usual circular selection rule for monolayers $\mathbf{p}_{cv}(\mathbf{q}) = \frac{p}{\sqrt{2}}[1\ i\tau]\delta_{\mathbf{q},0}$. DFT values for the optical matrix element, p, were taken from Ref. 1, and are close to the monolayer value for $MoSe_2$.[17]

The refractive index of the cavity medium enters our model via Maxwell's equations (so it must be evaluated at the optical frequency) and directly affects the cavity mode. A larger refractive index corresponds to a smaller resonant cavity length (i.e. light is squeezed down in size). We chose vacuum for simplicity in this work, $n_{bg} = 1$, but the polariton physics will be similar for other choices, just occurring at smaller cavity lengths.

S.1.5 Calculation details

Throughout our work, the end mirrors of the cavity are assumed equal and the reflectivity is set to a realistic frequency-independent value $r_m = -0.99$ (corresponding to a quality factor of 158 and a linewidth of 10.6 meV for a cavity tuned to $E^{(X)}$).[18] The exciton scattering loss is set to a constant value of $\hbar\Gamma = 1$ meV, which is appropriate for hBN-encapsulated monolayers at low temperatures below 100 K.[19] We have checked our results by comparing

our approach to the classical transfer-matrix method (see supplementary section S.3). We find an excellent agreement for the polariton dispersion and absorption calculated using the two methods. When using the Hopfield approach, only a single cavity mode is considered, this is valid due to the large free-spectral range relative to the exciton energy spacing for small cavity lengths.

S.2 Moiré Exciton Optics

The semi-classical exciton-light coupling,[20] (expressed within the minimal-coupling picture and dipole approximation) $\hat{H}_{\mathrm{XL}} = \frac{e}{m_0}\mathbf{A}_\sigma \cdot \sum_{\mathbf{k},\mathbf{q}} \mathbf{p}_{cv}(\mathbf{q})\hat{c}^\dagger_{\mathbf{k}+\mathbf{q}}\hat{v}_{\mathbf{k}} + \text{h.c.}$, can be written in the in the excitonic basis[2,11,16] (focusing only on the 1s exciton)

$$\hat{H}_{\mathrm{XL}} = A_\sigma \sum_{\mathbf{Q}} \Omega^{(X)}(\mathbf{Q})\hat{X}^\dagger_{\mathbf{Q}} + \text{h.c.} \quad \text{with} \quad \Omega^{(X)}(\mathbf{Q}) = \frac{e}{m_0}\mathbf{p}_{cv}(\mathbf{Q}) \cdot \mathbf{e}_\sigma \frac{1}{L}\sum_{\mathbf{k}} \psi(\mathbf{k}), \tag{S.4}$$

where $\mathbf{p}_{cv}(\mathbf{Q})$ is the optical matrix element, L is a normalization length, and $\mathbf{e}_\sigma$ the polarization vector of the light. Switching now to the moiré exciton basis, the light-exciton Hamiltonian reads[2,11]

$$\hat{H}_{\mathrm{YL}} = A_\sigma \sum_{\nu} \Omega^{(Y)}_\nu \hat{Y}^\dagger_\nu + \text{h.c.} \quad \text{with} \quad \Omega^{(Y)}_\nu = \sum_{i,j} \mathcal{C}_{\nu,ij}(\mathbf{Q}=0)\Omega^{(X)}\left(i\mathbf{g}_1 + j\mathbf{g}_2\right), \tag{S.5}$$

where only states within the light cone, $\mathbf{Q} = 0$, interact with light. The radiative coupling for moiré excitons can be expressed as

$$\gamma^{(Y)}_\nu = \frac{|\Omega^{(Y)}_\nu|^2}{2L^2\epsilon_0 n_{bg} c\hbar\omega}. \tag{S.6}$$

Typically, the frequency dependence is ignored,[16] and $\hbar\omega$ is set to the exciton energy. If we compare to the radiative coupling of the bilayer without moiré effects $\gamma^{(X)} = \frac{|\Omega^{(X)}|^2}{2L^2\epsilon_0 n_{bg} c\hbar\omega}$,

which is equivalent to equation (S.3) if $\hbar\omega$ is set equal to the exciton energy, we find

$$\gamma_\nu^{(Y)} = \left|\mathcal{C}_{\nu,i=j=0}(\mathbf{Q}=0)\right|^2 \gamma^{(X)}, \tag{S.7}$$

revealing that the oscillator strength (which is proportional to the radiative coupling) is conserved due to the normalisation of the moiré wavefunctions, i.e. $\sum_\nu \left|\mathcal{C}_{\nu,i=j=0}(\mathbf{Q}=0)\right|^2 = 1$.

S.2.1 Hopfield Method

The fully quantized exciton-light interaction for the 1s exciton in the absence of moiré effects can be written as

$$\hat{H}_{XL} = g^{(X)}\hat{X}^\dagger\hat{c} + \text{h.c} \quad \text{with} \quad g^{(X)} = \hbar\sqrt{\left(\frac{1+|r_m|}{|r_m|}\right)\frac{\gamma^{(X)}}{\tau}}. \tag{S.8}$$

The coupling term, $g^{(X)}$, is calculated by solving Maxwell's equations for a 2D excitonic layer centered in a Fabry-Perot cavity and approximating the cavity to be tuned close to the exciton energy.[21] Despite the approximation, we find excellent agreement with the T-matrix method (see supplementary section S.3), even away from the exciton energy. Transformation into the moiré exciton basis is simple, assuming the dipole approximation and neglecting the Q-dependence of $g^{(X)}$. Using Eq. (S.7), this results in the Eq. (2) of the main text.

A system of N excitons and one cavity photon is described by the Hamiltonian given by Eq. (1) of the main text. It can be diagonalised using the Hopfield approach.[22] By building a vector of operators $\mathbf{y} = \begin{bmatrix} \hat{c} & \hat{Y}_1 & \hat{Y}_2 & \dots & \hat{Y}_N \end{bmatrix}^T$, the Hamiltonian can be written

in a convenient form, $\mathbf{H} = \mathbf{y}^{\dagger}\mathbf{M}\mathbf{y}$, where

$$\mathbf{M} = \begin{bmatrix} E^{(c)} & g_1^* & g_2^* & \dots & g_N^* \\ g_1 & E_1^{(X)} & 0 & \dots & 0 \\ g_2 & 0 & E_2^{(X)} & \dots & 0 \\ \vdots & \vdots & \vdots & \ddots & \vdots \\ g_N & 0 & 0 & \dots & E_N^{(X)} \end{bmatrix}. \tag{S.9}$$

The matrix $\mathbf{M}$ is Hermitian and can diagonalised by a unitary transformation

$$\mathbf{H} = \mathbf{y}^{\dagger}\mathbf{U}\mathbf{U}^{-1}\mathbf{M}\mathbf{U}\mathbf{U}^{-1}\mathbf{y} = \boldsymbol{P}^{\dagger}\boldsymbol{\lambda}\boldsymbol{P}, \tag{S.10}$$

where the diagonal matrix $\boldsymbol{\lambda}$ gives the eigenvalues, the elements of $\mathbf{U}$ are the Hopfield coefficients, and the vector $\boldsymbol{P}(\boldsymbol{P}^{\dagger})$ gives the form of the creation(annihilation) operators of mixed light-exciton states

$$\boldsymbol{P} = \mathbf{U}^{-1}\mathbf{y} = \mathbf{U}^{\dagger}\mathbf{y} = \begin{bmatrix} \hat{P}_0 \\ \hat{P}_1 \\ \vdots \\ \hat{P}_N \end{bmatrix} = \begin{bmatrix} U_{00}^* & U_{10}^* & \dots & U_{N0}^* \\ U_{01}^* & U_{11}^* & \dots & U_{N1}^* \\ \vdots & \vdots & \ddots & \vdots \\ U_{0N}^* & U_{1N}^* & \dots & U_{NN}^* \end{bmatrix} \begin{bmatrix} \hat{c} \\ \hat{Y}_1 \\ \vdots \\ \hat{Y}_N \end{bmatrix} \tag{S.11}$$

This reveals that the polariton operators are $N+1$ linear combinations of the photon and excitons operators, weighted by the Hopfield coefficients

$$\hat{P}_m = \sum_{n=0}^{N} U_{nm}^* \hat{y}_n = U_{0m}^* \hat{c} + \sum_{\mu=1}^{N} U_{\mu m}^* \hat{Y}_{\mu}. \tag{S.12}$$

Here, the convention used is that the index 0 refers to the photonic contribution and $1 \to N$ to the N excitons. These relations can also be inverted to write the original photon and

exciton field operators in terms of the polariton operators, $\boldsymbol{P} = \mathbf{U}^{-1}\mathbf{y} \rightarrow \mathbf{y} = \mathbf{U}\boldsymbol{P}$, giving

$$\begin{aligned} \hat{c} &= \sum_{n=0}^{N} U_{0n}\hat{P}_n \\ \hat{Y}_\mu &= \sum_{n=0}^{N} U_{\mu n}\hat{P}_n. \end{aligned} \tag{S.13}$$

Throughout this work we treat excitons as non-interacting bosons and ignore their fermionic substructure, this is valid for low densities.[23] The Hopfield basis transformation preserves commutator relations, meaning that polaritons behave as bosons, and enforces useful orthogonality conditions on the Hopfield coefficients

$$\begin{aligned} [\hat{P}_n, \hat{P}_m^\dagger] &= \sum_{l=0}^{N} U_{ln}^* U_{lm} = \delta_{nm} \\ [\hat{Y}_\mu, \hat{Y}_\nu^\dagger] &= \sum_{n=0}^{N} U_{\mu n} U_{\nu n}^* = \delta_{\mu\nu}. \end{aligned} \tag{S.14}$$

S.3 Transfer Matrix Method

An alternative framework of a more classical flavour compared to the Hopfield method is the transfer(T)-matrix method. Here, the heterobilayer is modelled as a two-dimensional sheet current and a microscopic or phenomenological model is used to find the response function. This sort of technique has been applied to model the optics of graphene[24] and monolayer TMDs,[25] and has a long history in cavity polaritonics.[26] The advantage of this method is (i) its generality, for instance an arbitrary number of layers can be modelled, (ii) the ease in which experimentally relevant quantities such as reflection and absorption can be calculated, and (iii) the separation into distinct material (calculating the response function) and optics (solving Maxwell's equations) problems. In contrast, the Hopfield method is dependent on an analytical expression for the light-exciton coupling (or it can be treated as an external parameter), and the calculation of optical spectra requires a more complicated quantum Langevin approach (see Supplementary section S.5). A disadvantage of the T-

matrix method is that there is no access to the Hopfield coefficients, and it is difficult to extend to nonlinear physics such as polariton-polariton interaction. The two methods are equivalent in the limit of linear optics,[21] and we find excellent agreement for the polariton dispersion and absorption calculated using the two methods, see Fig. S1(a).

S.4 Photon-Induced Interlayer Hybridisation

We briefly remark that analysis of the Hopfield coefficients for the highest-energy polariton branch displayed in figure 1(c) of the main manuscript reveals that it predominately consists of the highest-energy moiré excitons from the $MoSe_2$ layer and the lowest-energy intralayer excitons located in the WSe_2 layer. This represents an interesting example of photon-induced *interlayer* hybridisation and is similar to what has been previously reported for untwisted MoS_2/WS_2 heterobilayers.[15] In the aforementioned reference, the coupling of the lowest-energy interlayer and intralayer was also explored. We found that, due to the small oscillator strength of the interlayer exciton and the large energy separation on the order of 300 meV with respect to the $MoSe_2$-based intralayer exciton, any hybridisation between the two was negligible.

S.5 Linear Optical Spectra of Polaritons

Cavity polaritons can couple to the outside universe via the non-zero transmission of the cavity mirrors. This can be modelled using the Heisenberg-Langevin equations along with the input-output relations,[27,28] which is valid for high-Q cavities (i.e. near-perfect end mirrors, $|r_m| \sim 1$). In this approach, the coupled internal cavity mode and the external radiation fields are quantized separately. This leads to a consistent description of both the radiative decay rate, and the coupling of the polaritons to input and output fields. The interaction between the cavity and external radiation modes is assumed to have a simple quadratic form and the rotating-wave approximation is used. It is also relatively straightforward to add

exciton loss in a similar fashion. Each exciton is coupled to a phonon bath, described by a phenomenological value $\hbar\Gamma_\nu$. The complete Hamiltonian for N excitons and one photon mode is

$$\begin{aligned}\hat{H} &= \sum_{\mu=1}^{N} E_\mu^{(Y)}\hat{Y}_\mu^\dagger\hat{Y}_\mu + E^{(c)}\hat{c}^\dagger\hat{c} + \sum_{\mu=1}^{N}\left[g_\mu\hat{Y}_\mu^\dagger\hat{c} + g_\mu^*\hat{Y}_\mu\hat{c}^\dagger\right] \\ &+ \sum_{i=L,R}\int_0^\infty d\omega\ \hbar\omega\hat{\mathcal{B}}_{i,\omega}^\dagger\hat{\mathcal{B}}_{i,\omega} + i\hbar\sum_{i=L,R}\int_{-\infty}^{\infty}\frac{d\omega}{2\pi}\ \sqrt{2\kappa_i(\omega)}\left[\hat{\mathcal{B}}_{i,\omega}^\dagger\hat{c} - \hat{\mathcal{B}}_{i,\omega}\hat{c}^\dagger\right] \\ &+ \sum_{\mu=1}^{N}\int_0^\infty d\omega\ \hbar\omega\hat{\mathcal{D}}_{\mu,\omega}^\dagger\hat{\mathcal{D}}_{\mu,\omega} + i\hbar\sum_{\mu=1}^{N}\int_{-\infty}^{\infty}\frac{d\omega}{2\pi}\ \sqrt{2\Gamma_\mu}\left[\hat{\mathcal{D}}_{\mu,\omega}^\dagger\hat{Y}_\mu - \hat{\mathcal{D}}_{\mu,\omega}\hat{Y}_\mu^\dagger\right],\end{aligned} \tag{S.15}$$

where the left-hand and right-hand external modes are described by the field operators $\hat{\mathcal{B}}_{i,\omega}$, $i = \{L, R\}$, and the phonon bath operator for *each* exciton is described by $\mathcal{D}_{\mu,\omega}$. The coupling parameters κ_L, κ_R and Γ_μ are frequency dependent in general, but we take the Markov approximation and assume that they have a flat frequency dependence in the energy ranges we are interested in. Furthermore, we take into account that $\omega \gg 1$ to set the lower limit of the frequency integration to minus infinity. Negative frequencies are nonphysical, but it is acceptable as we will deal with high frequencies and expect all the relevant physics to be close to the cavity frequency. With these assumptions, and using the expansion of the photon and exciton operators in Eq. (S.13), the total Hamiltonian can be written in the polariton basis as

$$\begin{aligned}\hat{H} &= \sum_{n=0}^{N} E_n^{(P)}\hat{P}_n^\dagger\hat{P}_n + \sum_{i=L,R}\int d\omega\ \hbar\omega\hat{\mathcal{B}}_{i,\omega}^\dagger\hat{\mathcal{B}}_{i,\omega} + \sum_{\mu=1}^{N}\int d\omega\ \hbar\omega\hat{\mathcal{D}}_{\mu,\omega}^\dagger\hat{\mathcal{D}}_{\mu,\omega} \\ &+ i\hbar\sum_{i=L,R}\sum_{n=0}^{N}\int_{-\infty}^{\infty}\frac{d\omega}{2\pi}\ \sqrt{2\kappa_i}\left(U_{0n}\hat{\mathcal{B}}_{i,\omega}^\dagger\hat{P}_n - U_{0n}^*\hat{\mathcal{B}}_{i,\omega}\hat{P}_n^\dagger\right) \\ &+ i\hbar\sum_{\mu=1}^{N}\int_{-\infty}^{\infty}\frac{d\omega}{2\pi}\ \sqrt{2\Gamma_\mu}\sum_{n=0}^{N}\left[U_{\mu n}\hat{\mathcal{D}}_{\mu,\omega}^\dagger\hat{P}_n - U_{\mu n}^*\hat{\mathcal{D}}_{\mu,\omega}\hat{P}_n^\dagger\right],\end{aligned} \tag{S.16}$$

where the left-hand and right-hand external modes are described by the field operators $\hat{\mathcal{B}}_{L,\omega}$ and $\hat{\mathcal{B}}_{R,\omega}$ respectively, and the phonon bath operator is described by $\hat{\mathcal{D}}_{\mu,\omega}$. The presence

of the Hopfield coefficients reflects the fact that only the photonic (excitonic) part of the polariton couples to the external radiation field (phonon baths).

The dynamics for the polariton and bath operators can be found from the Heisenberg equation of motion

$$
\begin{aligned}
i\hbar\frac{d}{dt}\hat{P}_n(t) &= E_n^{(P)}\hat{P}_n(t) - i\hbar U_{0n}^*\sum_{i=L,R}\int_{-\infty}^{\infty}\frac{d\omega}{2\pi}\ \sqrt{2\kappa_i}\hat{\mathcal{B}}_{i,\omega}(t) - i\hbar\sum_{\mu=1}^{N}U_{\mu n}^*\int_{-\infty}^{\infty}\frac{d\omega}{2\pi}\ \sqrt{2\Gamma_\mu}\hat{\mathcal{D}}_{\mu,\omega}(t)\\
i\hbar\frac{d}{dt}\hat{\mathcal{B}}_{i,\omega}(t) &= \hbar\omega\hat{\mathcal{B}}_{i,\omega}^{\dagger}(t) + i\hbar\sqrt{2\kappa_i}\sum_{n=0}^{N}U_{0n}\hat{P}_n(t)\\
i\hbar\frac{d}{dt}\hat{\mathcal{D}}_{\mu,\omega}(t) &= \hbar\omega\hat{\mathcal{D}}_{\mu,\omega} + i\hbar\sqrt{2\Gamma_\mu}\sum_{n=0}^{N}U_{\mu n}\hat{P}_n(t).
\end{aligned}
\tag{S.17}
$$

The dynamics of the reservoir operators can be solved by the usual prescription of first finding the formal solution in terms of an initial time $t_0 < t$,

$$
\begin{aligned}
\hat{\mathcal{B}}_{i,\omega}(t) &= \hat{\mathcal{B}}_{i,\omega}(t_0)e^{-i\omega(t-t_0)} + \sqrt{2\kappa_i}\sum_{n=0}^{N}U_{0n}\int_{t_0}^{t}\hat{P}_n(t')e^{-i\omega(t-t')}dt'\\
\hat{\mathcal{D}}_{\mu,\omega}(t) &= \hat{\mathcal{D}}_{\mu,\omega}(t_0)e^{-i\omega(t-t_0)} + \sqrt{2\Gamma_\mu}\sum_{n=0}^{N}U_{\mu n}\int_{t_0}^{t}\hat{P}_n(t')e^{-i\omega(t-t')}dt'.
\end{aligned}
\tag{S.18}
$$

These can then be substituted into the equation of motion for the polaritons. Thanks to the Markov approximation, a simple first-order differential equation in time is found to govern the polariton dynamics. In particular, if we assume the cavity to be only driven from one port,

$$
\frac{d}{dt}\hat{P}_n(t) = -i\frac{E_n^{(P)}}{\hbar}\hat{P}_n(t) - (\kappa_L+\kappa_R)\sum_{m=0}^{N}U_{0n}^*U_{0m}\hat{P}_m(t) - \sum_{\mu=1}^{N}\Gamma_\mu\sum_{m=0}^{N}U_{\mu n}^*U_{\mu m}\hat{P}_m(t) + U_{0n}^*\sqrt{2\kappa_L}a_{\rm in}(t),
\tag{S.19}
$$

where $a_{\rm in}(t) = -\int_{-\infty}^{\infty}\frac{d\omega}{2\pi}\,\hat{\mathcal{B}}_{L,\omega}(t_0)\exp[-i\omega(t-t_0)]$ is the input field and contains contributions from both vacuum quantum noise and a classical driving field.[27] As we are only interested in the mean field (large photon number), we drop all fluctuation terms (and drop the hats

on operators). Furthermore, we make the assumption that the phonon coupling term has a constant value for each moiré exciton, $\Gamma_\mu = \Gamma$. Using the orthogonality of the Hopfield coefficients (see Eq. (S.14)), one finds

$$\frac{d}{dt}P_n(t) = \left(-i\frac{E_n^{(P)}}{\hbar} - \Gamma\right)P_n(t) + U_{0n}^*\sqrt{2\kappa_L}a_{\rm in}(t) - U_{0n}^*(\kappa_L + \kappa_R - \Gamma)\sum_{m=0}^{N} U_{0m}P_m(t), \quad \text{(S.20)}$$

Boundary conditions at the mirrors are given by the input-output relations, allowing the input field to be related to the outgoing fields in each port.[27] These are calculated by solving the dynamics for the reservoir operators in terms of a future time $t_1 > t$ to give the time-reversed polariton dynamics and introducing the outgoing fields $a_{\rm ref} = +\int_{-\infty}^{\infty}\frac{d\omega}{2\pi}\,\mathcal{B}_{L,\omega}(t_1)\exp[-i\omega(t-t_1)]$ and $a_{\rm tran} = -\int_{-\infty}^{\infty}\frac{d\omega}{2\pi}\,\mathcal{B}_{R,\omega}(t_1)\exp[-i\omega(t-t_1)]$. For a system with time-reversal symmetry, the two dynamical equations can be set equal and the input-output relations are found by closing off each port in turn. The presence of material loss in the model due to the phonon bath complicates matters and means one must be careful how to apply this procedure to the dynamics of the phonon reservoir operator. As the only contribution of phonons is to add a loss channel, we employ a restricted time reversal approach[29] where lossy materials remain lossy under time reversal. In other words, only Maxwell's equations are reversed and the microscopic processes coupled to the electromagnetic field are unchanged. In our case this means that the dynamics of $\hat{\mathcal{D}}_{\mu,\omega}(t)$ remain the same under time reversal and we obtain the following input-output relations

$$a_{\rm ref}(t) = -a_{\rm in}(t) + \sqrt{2\kappa_L}\sum_n U_{0n}P_n(t), \quad a_{\rm trans}(t) = \sqrt{2\kappa_R}\sum_n U_{0n}P_n(t), \quad \text{(S.21)}$$

i.e. the form of the input-output relations is unchanged by the presence of the phonon reservoir. This approach is consistent with how material loss is added within the well-known coupled-mode theory commonly used in photonics.[30,31] In fact, the coupled-mode theory can be viewed as a macroscopic limit of the quantum Langevin plus input-output equations.

In the next step, Eq. (S.20) and (S.21) can both be Fourier transformed and combined

to give the reflection and transmission coefficients

$$\begin{aligned} r(\omega) &= \frac{a_{\mathrm{ref}}(\omega)}{a_{\mathrm{in}}(\omega)} = \frac{-1 + (\kappa_L - \kappa_R + \Gamma)\Pi(\omega)}{1 + (\kappa_L + \kappa_R - \Gamma)\Pi(\omega)} \\ it(\omega) &= \frac{a_{\mathrm{trans}}(\omega)}{a_{\mathrm{in}}(\omega)} = \frac{\sqrt{2\kappa_R}\sqrt{2\kappa_L}\Pi(\omega)}{1 + (\kappa_L + \kappa_R - \Gamma)\Pi(\omega)}, \end{aligned} \tag{S.22}$$

with $\Pi(\omega) = \sum_{n=0}^{N} \frac{|U_{0n}|^2}{i\left(E_n^{(P)}/\hbar - \omega\right) + \Gamma}$. It can be checked that Eq. (S.22) correctly describes a bare Fabry-Perot cavity in the limit of a pure photonic mode, i.e. $|U_{0n}| = 1$. Furthermore, in the case of a single polariton Eq. (S.22) reduces to

$$\begin{aligned} it(\omega) &= -\frac{\sqrt{2\kappa_R}\sqrt{2\kappa_L}|U_0|^2}{i(\omega^{(P)} - \omega) + (\kappa_L + \kappa_R)|U_0|^2 + \Gamma|U_1|^2} \\ r(\omega) &= \frac{-i(\omega^{(P)} - \omega) + (\kappa_L - \kappa_R)|U_0|^2 - \Gamma|U_1|^2}{i(\omega^{(P)} - \omega) + (\kappa_L + \kappa_R)|U_0|^2 + \Gamma|U_1|^2}, \end{aligned} \tag{S.23}$$

which agrees with what is found directly using the coupled-mode theory for a single mode interacting with two ports,[30,31] but with the magnitude of photonic and material decay rate determined by the Hopfield coefficients.

The only task remaining is to determine the coupling constants κ_L and κ_R. By using the exact analytical expression for a lossless and symmetric bare Fabry-Perot cavity, it is possible to expand for frequencies close to the cavity frequency and a high-quality cavity[32] ($T_m \approx 0$), and compare to Eqs. (S.22) in the purely photonic limit to find $\kappa_L = \kappa_R = cT_m/(4L)$.

It is straightforward to find an expression for the absorption from energy conservation $A(\omega) = 1 - |r(\omega)|^2 - |it(\omega)|^2$. Taking a symmetric cavity, $\kappa = \kappa_L = \kappa_R$, and using Eqs. (S.22) gives

$$A(\omega) = \frac{4\kappa(\Re[\Pi(\omega)] - \Gamma|\Pi(\omega)|^2)}{|1 + (2\kappa - \Gamma)\Pi(\omega)|^2}. \tag{S.24}$$

It is instructive to make further simplifications to find an approximate analytical expression for the absorption. Inspired by the similar derivation of the Elliot formula,[11,16] we expand the numerator and denominator, and neglect interference effects. This is justified for energetically well-separated moiré excitons relative to the scattering rate $\hbar\Gamma$. After some simple

algebra, this returns Eq. (3) of the main text,

$$A(\omega) = \sum_{n=0}^{N} \frac{4\tilde{\gamma}_n \tilde{\Gamma}_n}{(\omega - E_n^{(P)}/\hbar)^2 + (2\tilde{\gamma}_n + \tilde{\Gamma}_n)^2}, \tag{S.25}$$

which has a remarkable similarity with the conventional Elliot formula. While it does not include light-matter fully self-consistently like Eq. (S.24), we find an excellent agreement with the T-matrix method. This is demonstrated in Fig. S1(a).

S.6 Polaritonic Critical Coupling Condition

Here we discuss the conditions for peak absorption in Eq. (S.25) (Eq. (3) of the main text). Focusing only on the polariton branch n and evaluating the equation at $\hbar\omega = E_n^{(P)}$ gives

$$A = \frac{4\tilde{\gamma}_n \tilde{\Gamma}_n}{(2\tilde{\gamma}_n + \tilde{\Gamma}_n)^2}. \tag{S.26}$$

The maximum absorption of 0.5 is reached at the condition $2\tilde{\gamma}_n = \tilde{\Gamma}_n$. The value of the Hopfield coefficients that satisfy this condition depend on the ratio between the exciton scattering rate, Γ, and the cavity decay rate κ. For the specific case where the non-radiative decay rate, Γ, is balanced by the total radiative decay through both ports of the bare cavity, 2κ, the maximum absorption is achieved for an equal light-matter contribution to the polariton, i.e. $|U_{0n}|^2 = 0.5$. For the parameters chosen throughout this work, $\Gamma = 1$ meV and $|r_m| = 0.99$, the critical coupling condition for the P_1 polariton at a 1° twist angle is found at the cavity length $L_{\max} = 364$ nm (vertical black-dotted line), which corresponds to a Hopfield coefficient of $|U_{0n}|^2 = 0.16$, cf. Fig. S1(a). For more insight, we show in Fig. S1(b) a plot of the effective decay rates, as well as the corresponding bare-cavity decay rate and exciton scattering rate, as a function of the cavity length. We can observe that the critical coupling condition occurs (crossing point of the blue- and red-solid lines) at the peak absorption cavity length of $L_{\max}$ (vertical black-dotted line).

S.7 Polariton Group Velocity and Effective Mass

To derive an expression for the polariton group velocity for a general number of excitons, we start by evaluating the commutator relation $[\hat{P}_n, \hat{H}]$ twice: once for the Hamiltonian expressed in the diagonal form with polariton operators, and then expressed in the original form with exciton and photon operators. This gives two equations

$$\begin{aligned}\left(E_n^{(P)}(\mathbf{k}_\parallel) - E^{(c)}(\mathbf{k}_\parallel)\right) U_{0n}(\mathbf{k}_\parallel) &= \sum_{\nu=1}^{N} g_\nu(\mathbf{k}_\parallel) U_{\nu n}(\mathbf{k}_\parallel) \\ \left(E_n^{(P)}(\mathbf{k}_\parallel) - E_\nu^{(Y)}(\mathbf{k}_\parallel)\right) U_{\nu n}(\mathbf{k}_\parallel) &= g_\nu(\mathbf{k}_\parallel) U_{0n}(\mathbf{k}_\parallel),\end{aligned} \tag{S.27}$$

where $E^{(c)}$ is the cavity photon energy. The second equation can be plugged into the first equation resulting in

$$\left(E_n^{(P)}(\mathbf{k}_\parallel) - E^{(c)}(\mathbf{k}_\parallel)\right) = \sum_{\nu=1}^{N} \frac{g_\nu^2(\mathbf{k}_\parallel)}{E_n^{(P)}(\mathbf{k}_\parallel) - E_\nu^{(Y)}(\mathbf{k}_\parallel)}. \tag{S.28}$$

This expression can be differentiated with respect to in-plane momentum, and rearranged to give the group velocity for the nth polariton

$$v_n^{(P)}(\mathbf{k}_\parallel) = \left|U_{0n}(\mathbf{k}_\parallel)\right|^2 v^{(c)}(\mathbf{k}_\parallel) + \sum_{\nu=1}^{N} \left|U_{\nu n}(\mathbf{k}_\parallel)\right|^2 v_\nu^{(Y)}(\mathbf{k}_\parallel) + 2U_{0n}(\mathbf{k}_\parallel) \sum_{\nu=1}^{N} U_{\nu n}^*(\mathbf{k}_\parallel) \partial_{\mathbf{k}_\parallel} g_\nu(\mathbf{k}_\parallel)/\hbar \tag{S.29}$$

This is a generalisation of the expression for the group velocity found in Ref. 33 for a single type of exciton and photon. For momenta close to $\mathbf{k}_\parallel = 0$, the first derivative of the coupling will be small and third term can be ignored. In fact, this is exact for TM-polarized cavity modes, where the coupling is constant with in-plane momentum.[34] For an isotropic dispersion, which is valid at the γ point of the exciton dispersion, the polariton effective mass can then be found from the group velocity using

$$m_n^{(P)} = \frac{\hbar}{\partial_{\mathbf{k}_\parallel} v_n^{(P)}(\mathbf{k}_\parallel)|_{\mathbf{k}_\parallel=0}}, \tag{S.30}$$

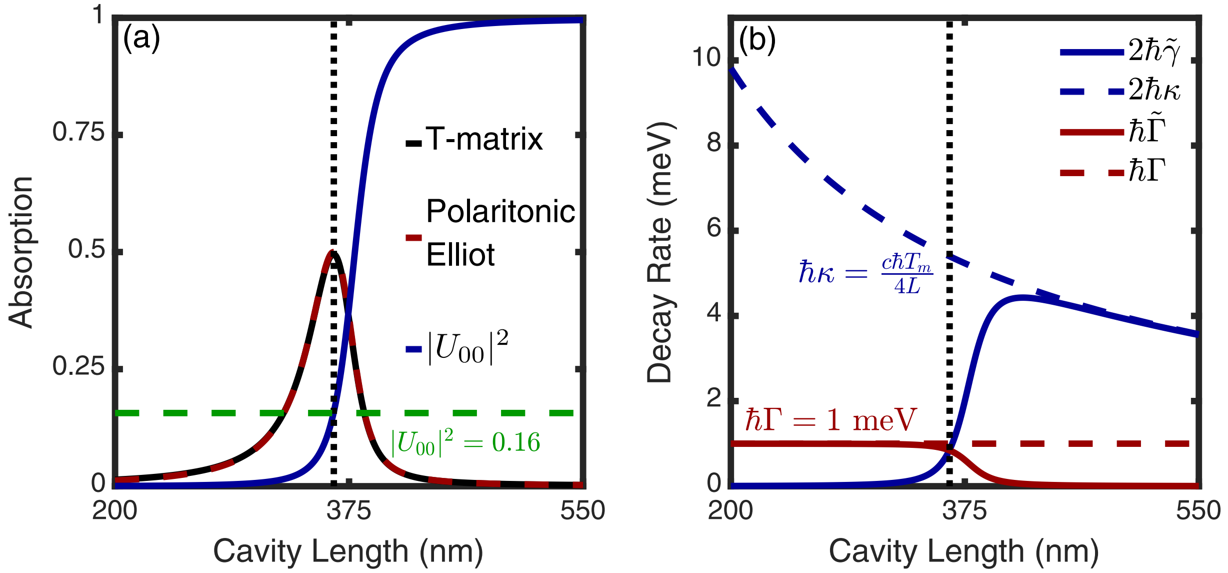

Figure S1: (a) Resonant absorption for the P_1 polariton at a 1° twist angle calculated with the T-matrix method (black line) and the polaritonic Elliot formula (Eq. (S.25), dashed-red line). The Hopfield coefficient describing the photonic contribution (U_{00}, blue line) is also shown. The peak absorption of 0.5 is indicated by the vertical black-dotted line, and the corresponding value of the Hopfield coefficient that gives this condition is indicated by the dashed-green line. (b) Comparison of twice the effective radiative coupling (blue line), and the effective scattering rate (red line), against cavity length. The bare-cavity decay rate and exciton scattering rate are shown by the blue- and red-dashed lines respectively. The peak absorption (vertical black-dotted line) is given by the polaritonic critical coupling condition of $2\tilde{\gamma} = \tilde{\Gamma}$.

which, combined with equation S.29, gives Eq. (4) of the main text. Note that we can ignore the derivatives of the Hopfield coefficients as the first derivative vanishes at $\mathbf{k}_{\parallel} = 0$, reflecting the even symmetry at this point.

References

(1) Ovesen, S.; Brem, S.; Linderälv, C.; Kuisma, M.; Korn, T.; Erhart, P.; Selig, M.; Malic, E. Interlayer exciton dynamics in van der Waals heterostructures. *Communications Physics* **2019**, *2*, 1–8.

(2) Brem, S.; Linderälv, C.; Erhart, P.; Malic, E. Tunable phases of moiré excitons in van der Waals heterostructures. *Nano letters* **2020**, *20*, 8534–8540.

(3) Rosenberger, M. R.; Chuang, H.-J.; Phillips, M.; Oleshko, V. P.; McCreary, K. M.; Sivaram, S. V.; Hellberg, C. S.; Jonker, B. T. Twist angle-dependent atomic reconstruction and moiré patterns in transition metal dichalcogenide heterostructures. *ACS nano* **2020**, *14*, 4550–4558.

(4) Seyler, K. L.; Rivera, P.; Yu, H.; Wilson, N. P.; Ray, E. L.; Mandrus, D. G.; Yan, J.; Yao, W.; Xu, X. Signatures of moiré-trapped valley excitons in MoSe 2/WSe 2 heterobilayers. *Nature* **2019**, *567*, 66–70.

(5) Srivastava, A.; Sidler, M.; Allain, A. V.; Lembke, D. S.; Kis, A.; Imamoğlu, A. Optically active quantum dots in monolayer WSe2. *Nature nanotechnology* **2015**, *10*, 491–496.

(6) Li, W.; Lu, X.; Dubey, S.; Devenica, L.; Srivastava, A. Dipolar interactions between localized interlayer excitons in van der Waals heterostructures. *Nature Materials* **2020**, *19*, 624–629.

(7) Bistritzer, R.; MacDonald, A. H. Moiré bands in twisted double-layer graphene. *Proceedings of the National Academy of Sciences* **2011**, *108*, 12233–12237.

(8) Wu, F.; Lovorn, T.; MacDonald, A. H. Topological exciton bands in moiré heterojunctions. *Physical review letters* **2017**, *118*, 147401.

(9) Wu, F.; Lovorn, T.; MacDonald, A. Theory of optical absorption by interlayer excitons in transition metal dichalcogenide heterobilayers. *Physical Review B* **2018**, *97*, 035306.

(10) Yu, H.; Liu, G.-B.; Tang, J.; Xu, X.; Yao, W. Moiré excitons: From programmable quantum emitter arrays to spin-orbit–coupled artificial lattices. *Science advances* **2017**, *3*, e1701696.

(11) Brem, S. Microscopic Theory of Exciton Dynamics in Two-Dimensional Materials. **2020**,

(12) Laturia, A.; Van de Put, M. L.; Vandenberghe, W. G. Dielectric properties of hexagonal boron nitride and transition metal dichalcogenides: from monolayer to bulk. *npj 2D Materials and Applications* **2018**, *2*, 1–7.

(13) Rivera, P.; Schaibley, J. R.; Jones, A. M.; Ross, J. S.; Wu, S.; Aivazian, G.; Klement, P.; Seyler, K.; Clark, G.; Ghimire, N. J., et al. Observation of long-lived interlayer excitons in monolayer MoSe 2–WSe 2 heterostructures. *Nature communications* **2015**, *6*, 1–6.

(14) Nagler, P.; Plechinger, G.; Ballottin, M. V.; Mitioglu, A.; Meier, S.; Paradiso, N.; Strunk, C.; Chernikov, A.; Christianen, P. C.; Schüller, C., et al. Interlayer exciton dynamics in a dichalcogenide monolayer heterostructure. *2D Materials* **2017**, *4*, 025112.

(15) Latini, S.; Ronca, E.; De Giovannini, U.; Hübener, H.; Rubio, A. Cavity control of excitons in two-dimensional materials. *Nano letters* **2019**, *19*, 3473–3479.

(16) Kira, M.; Koch, S. Many-body correlations and excitonic effects in semiconductor spectroscopy. *Progress in quantum electronics* **2006**, *30*, 155–296.

(17) Dufferwiel, S.; Schwarz, S.; Withers, F.; Trichet, A.; Li, F.; Sich, M.; Del Pozo-Zamudio, O.; Clark, C.; Nalitov, A.; Solnyshkov, D., et al. Exciton–polaritons in van der Waals heterostructures embedded in tunable microcavities. *Nature communications* **2015**, *6*, 1–7.

(18) Zhang, L.; Wu, F.; Hou, S.; Zhang, Z.; Chou, Y.-H.; Watanabe, K.; Taniguchi, T.; Forrest, S. R.; Deng, H. Van der Waals heterostructure polaritons with moiré-induced nonlinearity. *Nature* **2021**, *591*, 61–65.

(19) Ajayi, O. A.; Ardelean, J. V.; Shepard, G. D.; Wang, J.; Antony, A.; Taniguchi, T.; Watanabe, K.; Heinz, T. F.; Strauf, S.; Zhu, X., et al. Approaching the intrinsic photoluminescence linewidth in transition metal dichalcogenide monolayers. *2D Materials* **2017**, *4*, 031011.

(20) Haug, H.; Koch, S. W. *Quantum theory of the optical and electronic properties of semiconductors*; World Scientific Publishing Company, 2009.

(21) Kavokin, A.; Malpuech, G. *Cavity Polaritons*; Thin films and Nanostructures; Elsevier, 2003; pp 29–45.

(22) Hopfield, J. Theory of the contribution of excitons to the complex dielectric constant of crystals. *Physical Review* **1958**, *112*, 1555.

(23) Erkensten, D.; Brem, S.; Malic, E. Exciton-exciton interaction in transition metal dichalcogenide monolayers and van der Waals heterostructures. *Physical Review B* **2021**, *103*, 045426.

(24) Zhan, T.; Shi, X.; Dai, Y.; Liu, X.; Zi, J. Transfer matrix method for optics in graphene layers. *Journal of Physics: Condensed Matter* **2013**, *25*, 215301.

(25) Vasilevskiy, M. I.; Santiago-Perez, D. G.; Trallero-Giner, C.; Peres, N. M.; Kavokin, A. Exciton polaritons in two-dimensional dichalcogenide layers placed in a planar microcavity: Tunable interaction between two Bose-Einstein condensates. *Physical Review B* **2015**, *92*, 245435.

(26) Savona, V.; Andreani, L.; Schwendimann, P.; Quattropani, A. Quantum well excitons in semiconductor microcavities: Unified treatment of weak and strong coupling regimes. *Solid State Communications* **1995**, *93*, 733–739.

(27) Collett, M.; Gardiner, C. Squeezing of intracavity and traveling-wave light fields produced in parametric amplification. *Physical Review A* **1984**, *30*, 1386.

(28) Gardiner, C. W.; Collett, M. J. Input and output in damped quantum systems: Quantum stochastic differential equations and the master equation. *Physical Review A* **1985**, *31*, 3761.

(29) Asadchy, V. S.; Mirmoosa, M. S.; Díaz-Rubio, A.; Fan, S.; Tretyakov, S. A. Tutorial on electromagnetic nonreciprocity and its origins. *Proceedings of the IEEE* **2020**, *108*, 1684–1727.

(30) Haus, H. Waves and fields in optoelectronics. *Prentice-Hall, Englewood Cliffs* **1984**,

(31) Suh, W.; Yanik, M.; Solgaard, O.; Fan, S. Displacement-sensitive photonic crystal structures based on guided resonance in photonic crystal slabs. *Applied physics letters* **2003**, *82*, 1999–2001.

(32) Németh, N.; White, D.; Kato, S.; Parkins, S.; Aoki, T. Transfer-Matrix Approach to Determining the Linear Response of All-Fiber Networks of Cavity-QED Systems. *Physical Review Applied* **2020**, *13*, 064010.

(33) Freixanet, T.; Sermage, B.; Tiberj, A.; Planel, R. In-plane propagation of excitonic cavity polaritons. *Physical Review B* **2000**, *61*, 7233.

(34) Panzarini, G.; Andreani, L. C.; Armitage, A.; Baxter, D.; Skolnick, M.; Astratov, V.; Roberts, J.; Kavokin, A. V.; Vladimirova, M. R.; Kaliteevski, M. Cavity-polariton dispersion and polarization splitting in single and coupled semiconductor microcavities. *Physics of the Solid State* **1999**, *41*, 1223–1238.
